# Glioblastoma Stem Cells and Tumour Microenvironment: Interactions Across Hypoxia, Vasculature and Immune Modulation

**DOI:** 10.3390/ijms27062557

**Published:** 2026-03-11

**Authors:** Karina Biserova, Ilze Strumfa

**Affiliations:** 1 Department of Pathology, Riga Stradins University, 16 Dzirciema Street, LV-1007 Riga, Latvia; ilze.strumfa@rsu.lv; 2 Pathology Institute, Riga Paul Stradins Clinical University Hospital, 13 Pilsonu Street, LV-1002 Riga, Latvia

**Keywords:** cancer stem cells, glioblastoma, microenvironment, cancer

## Abstract

Glioblastoma (GBM) is an aggressive brain tumour known for its ability to resist the current treatment protocols. A major reason for this resistance is a minor group of cells within the tumour called glioblastoma stem cells (GSCs). These cells drive tumour growth, invasion, and recurrence after therapy. GSCs survive and expand within a specific microenvironment that protects and supports them. Three of the most important niches are: hypoxic (low oxygen) regions, which trigger survival pathways and make GSCs more resistant to treatment; perivascular areas near blood vessels, which provide nutrients and signals that maintain stem-like properties; and immune-related zones, where inflammatory and suppressive signals help GSCs escape the body’s defences. Together, these environments allow GSCs to thrive and contribute to the tumour’s persistence. This review highlights how hypoxia, blood vessel niches, and immune interactions work together to sustain GSCs and promote GBM progression. A clearer understanding of these supportive environments may lead to new treatment approaches aimed at disrupting GSC survival and improving patient outcomes.

## 1. Introduction

Glioblastoma represents one of the most deadly malignancies in humans [1]. In the most recent edition of the WHO classification of central nervous system tumours, glioblastoma is defined as grade 4 tumour, featuring unmutated or wildtype gene of isocitrate dehydrogenase (IDH) [2]. Glioblastoma is managed by surgical resection of the tumour mass followed by radiation and chemotherapy with temozolamide [3].

Despite the advances in standard treatment protocols, survival of the patients remains grim. Most individuals diagnosed with GBM have a median survival of only 14 months, with long-term survival (over five years) achieved by less than 10% of patients [4]. This underlines the urgent need to better understand the underlying biological mechanisms driving tumour growth, recurrence, and resistance to current therapies.

Glioblastoma stem cells—pluripotent tumour-initiating cells—are considered to be one of the key factors in glioblastoma viability and treatment resistance [5]. The therapeutic inefficacy in treating GBM is primarily due to its ability to infiltrate surrounding healthy brain tissues, blood–brain barrier, immune evasion and other complex treatment resistance mechanisms. In recent years despite molecular typing of glioblastoma tumour cells, tumour microenvironment (TME) is considered one of the crucial players in tumorigenesis and resistance to treatment, as well as a promising therapeutic target [6,7].

GBM cells engage with both cellular (such as glial cells, endothelial cells, and glioblastoma stem cells) and non-cellular components (such as the extracellular matrix and its produced molecules) of the tumour environment. Together, these elements shape tumour’s invasive and adaptive properties, fuelling its progression and making it difficult to treat effectively [8].

Important qualities of tumour-initiating microenvironment include hypoxia, which significantly supports GSC maintenance [9]. Secondly, malignant growth is highly dependent on vascular proliferation, primarily driven by vascular endothelial growth factor-A [10]. Thirdly, glioblastoma is characterized by substantial quantity of immune cell infiltrates, propagating pro-cancerogenic qualities of tumour environment [11].

Understanding the unique biology of glioblastoma and its stem cell population in combination with microenvironmental factors is foundational to addressing the disease’s notorious resistance to treatment. In this review we describe three microenvironmental domains—hypoxia, vascular niche, and immune modulation—that support and sustain GSCs, driving tumour viability and treatment resistance.

## 2. Hypoxia—A Fertile Ground for GSC Persistence

Glioblastomas are known to be notably hypoxic, with oxygen levels in tumour tissue significantly lower than those found in ambient air or in healthy tissues. While atmospheric oxygen concentration is about 20.8%, normal physiological oxygen levels in adult human tissues range between 2% and 9%. In glioblastomas, these levels can drop far below this range, setting the stage for significant metabolic and molecular changes in the tumour cells [12]. Stem cell viability and functionality under hypoxic circumstances have also been efficiently studied. In early studies, embryonic stem cells were found to grow equally well when cultured in normoxic (20% oxygen (O_2_) or hypoxic conditions (1% O_2_) during the first five days. Regarding the functionality, embryonic stem cells showed increased expression of endothelial growth factor (VEGF) mRNA under hypoxic conditions in an in vitro model. The upregulation of VEGF was in accordance with the expected physiological reaction to low oxygen levels by modulating the expression of certain genes [13]. In physiological processes, hypoxia and hypoxia-inducible factors (HIFs) have an important beneficial role in the stem cell differentiation into endoderm, pulmonary epithelial cells, as well as neural crest [14,15]. However, appropriate timing and extent of hypoxia, or appropriate level of HIF expression, is mandatory to ensure correct embryogenesis. The physiological impact of hypoxia on embryonic stem cells and the related molecular regulatory mechanisms become reflected in the origin and survival of glioblastoma stem cells [14,15].

Glioblastoma stem cells (GSCs) recognized by the expression of the stem cell marker CD133 not only survive hypoxic stress but actually expand under such conditions, reinforcing the idea that hypoxia acts selectively, favouring survival of the most treatment-resistant cells [16]. Observing glioblastoma stem cells (GSCs) under hypoxic conditions can give new insights into glioblastoma research and treatment possibilities.

Hypoxia-inducible factors (HIFs), mostly HIF1α and HIF2α, play central roles in orchestrating the genetic response to tissue oxygen deprivation. These transcription factors bind to hypoxia-responsive elements (HREs) in DNA to activate genes involved in angiogenesis (e.g., vascular endothelial growth factor VEGF), metabolism (e.g., glucose transporter 1 GLUT1), and stemness (e.g., octamer-binding transcription factor OCT4). GLUT1 is a key protein, ensuring transmembranous glucose uptake into the cells [17]. Besides upregulation of GLUT1 in hypoxia, elevated levels have been found in a range of pathological conditions. In allergic rhinitis, upregulation of GLUT1 contributes to epithelial barrier dysfunction [18]. GLUT1 is also upregulated in a wide spectrum of tumours, either via hypoxic mechanisms or as a manifestation of aerobic glycolysis—the Warburg effect, and frequently has negative prognostic impact [17]. HIF1α is among the major inducers of GLUT1 [19]. HIF2α, in particular, has been shown to upregulate OCT4—a transcription factor—that is a crucial regulator of self-renewal and pluripotency in both normal and cancer stem cells [20,21]. Therefore, HIFs represent a promising therapeutic target for different types of cancers [22].

An additional mechanism, ensuring cell sustainability and proliferation, is hexokinase 2 (HK2) expression initiated by HIF-1α. HK2 is an enzyme that utilizes ATP, regulating glucose phosphorylation and further utilization for metabolic means. In cancer, under hypoxic conditions, production of HK2 is increased and prevents cytochrome c release from the mitochondria, consequently inhibiting apoptosis of cells [23].

Although standard cancer treatments target oxygen-rich proliferating cells, no therapies yet specifically target hypoxic tumour regions, even though emerging clinical and experimental evidence underscores that hypoxia-driven HIF signalling is a key driver of cancer progression. HIF-driven processes have promising potential of developing HIF-targeted cancer therapies [22].

**Figure 1:**
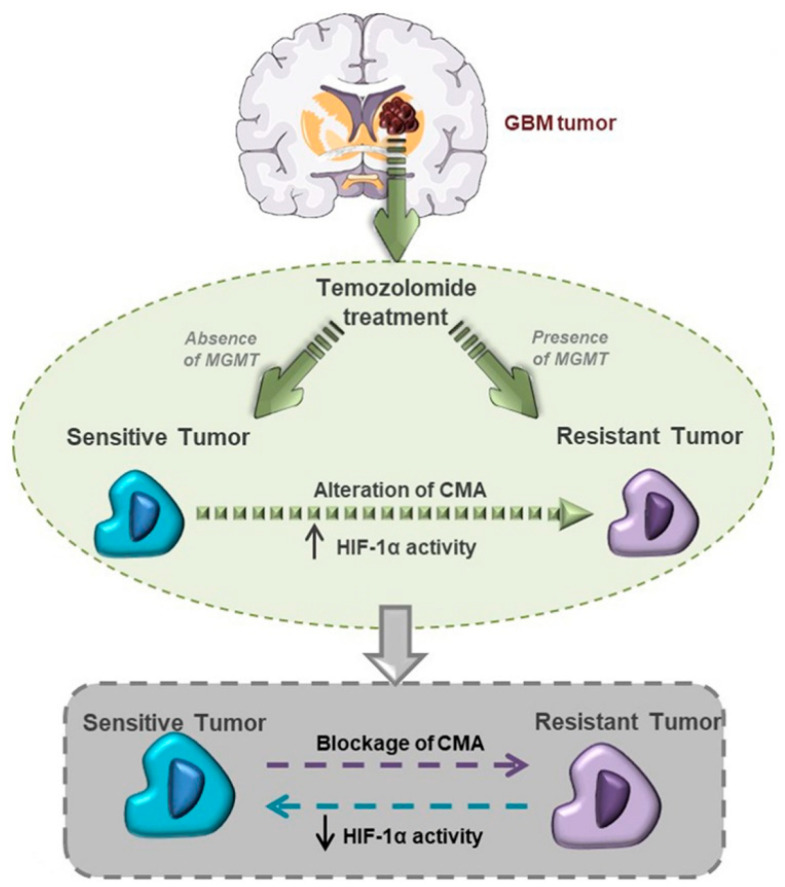
Higher hypoxia-inducible factor 1a (HIF1a) activity alters tumour cell (HIF)-1α/chaperone-mediated autophagy (CMA) and decreases cell responsiveness to temozolomide (TMZ). Copyright © 2018 Lo Dico, Martelli, Diceglie, Lucignani and Ottobrini. Figure replicated from Lo Dico et al., 2018 [28] under the terms and conditions of the Creative Commons Attribution License (CC BY), provided at https://creativecommons.org/licenses/by/4.0/ (accessed on 1 February 2026). Changes made: Figure legend.

Hypoxic conditions also maintain resistance against the treatment. Nowadays, the standard of care for glioblastoma includes surgical resection, followed by radiotherapy and chemotherapy with temozolomide: an alkylating agent of the imidazotetrazine class capable of crossing the blood–brain barrier. The cytotoxic effect of temozolamide is ensured by DNA methylation in N7-guanine position (70%), followed by the N3-adenine (9%) and O6-guanine (5%) residue, but it can be reversed via demethylation by O6-methylguanine-DNA methyltransferase (MGMT) [24,25,26]. Tumours showing high levels of MGMT are more resistant against temozolamide, while epigenetic inactivation of MGMT through promotor methylation is associated with better therapeutic efficacy. Importantly, MGMT methylation status is not a feature limited to the bulk of glioblastoma, composed by proliferating tumour cells. Instead, MGMT promoter methylation status in glioblastoma stem cells remains unchanged during differentiation to daughter tumour cell progeny and is comparable between glioblastoma stem cell cultures and surgically resected tumour tissues [27]. In hypoxic conditions, HIF1a shows activity in determining sensitivity or resistance of tumour cells towards temozolomide. Lo Dico et al. highlighted the silencing effect on cell responsiveness to treatment when evaluating apoptosis-related genes and glioblastoma cell viability. Higher HIF1a activity alters tumour cell (HIF)-1α/chaperone-mediated autophagy (CMA) and decreases cell responsiveness to temozolomide (TMZ) [28] (Figure 1).

Hypoxia also boosts the expression of receptor for advanced glycation end products (RAGE), an alarmin receptor linked to sensing inflammatory cues; importantly, this increase is suppressed when HIF-1α is inhibited by agents like digoxin or acriflavine. When exposed to necrotic cell extracts, which mimic inflammation, GSCs display enhanced invasion and adhesion behaviours, particularly under hypoxic conditions. These pro-migratory and adhesive effects are mitigated by HIF-1α inhibitors. In vivo, GSCs implanted into one hemisphere of immunodeficient mouse brains migrate toward the opposite hemisphere if injected there with necrotic material, indicating that necrosis-induced inflammation is a potent attractant for these cells [29].

Another pathogenetic mechanism of GSC upregulation in hypoxic conditions in the tumour core is mediated via transforming growth factor beta-induced protein (TGFBI) that is secreted by M2-polarized tumour-associated macrophages (TAMs). In malignant tumours, macrophages can differentiate into pro-inflammatory M1 subtype, which is considered to act mainly against the tumour progression, and immunosuppressive, pro-angiogenetic, pro-tumorous M2 subtype, associated with adverse prognosis [30,31]. In hypoxia, HIF1a upregulates TGFBI in glioblastoma stem cells. Increased levels of TGFBI correlate both with expression of stem cell markers sex determining region Y-box transcription factor 2 (SOX2), prominin-1 (CD133) and oligodendrocyte transcription factor 2 (OLIG2) and with functional manifestations of stemness, such as sphere formation, viability, proliferation and escape from apoptosis. The pathogenetic mechanisms of TGFBI-induced stem cell differentiation include activation of AKT-*c-MYC* signalling pathway in GSCs and interaction between TGFBI and ephrin receptor A2 (EphA2). The PI3K/AKT/mTOR/MYC molecular cascade is a highly conserved signal transduction network in eukaryotic cells that promotes survival, growth, and proliferation [32]. This pathway is upregulated in many malignant tumours [32], including glioblastoma, showing activation of this pathway in approximately 86% of cases [33,34]. In glioblastoma, the consequences include the classic hallmarks of cellular proliferation and motility, as well as maintenance of glioblastoma stem cells [33,35,36]. TGFBI also binds to ephrin receptor A2 (EphA2). This interaction prevents EphA2 protein from degradation and promotes the self-renewal of GSCs. Ligand-independent activation of EphA2 in glioblastoma is associated with MEK/ERK/RSK signalling pathway and leads to increased cell migration, invasion and proliferation [37]. This highlights how hypoxia is not only a stressor but also a driver of tumour-supportive interactions between GSCs and immune cells [38,39].

Another key adaptation to hypoxia involves long non-coding RNAs (lncRNAs). One such lncRNA—lung cancer-associated transcript 1 (LUCAT1), is regulated by the hypoxia-inducible factor HIF1α. LUCAT1 contributes to a positive feedback loop that strengthens the hypoxia response, enhances the self-renewal of GSCs, and accelerates tumour growth [40].

In addition to LUCAT1, galectin-8 has also been implicated in the survival of GSCs under hypoxia. This protein supports autophagy and the preservation of stem cell properties. Inhibiting galectin-8 has been shown to reduce the stemness of GSCs, suggesting it may be a promising therapeutic target [41].

Transgelin, another molecule activated under hypoxia, helps GSCs withstand low oxygen levels by modulating the acetylation of the p53 protein—a crucial factor in cell cycle control and apoptosis [42].

In 3D cultures derived from patient tumours, hypoxia was shown to significantly boost GSC invasiveness and induce the expression of genes linked to glucose metabolism, angiogenesis, immune response, and autophagy. Notably, IDH-mutant and IDH-wildtype gliomas respond differently to hypoxic stress: the former shifts toward an astrocytic phenotype that could be considered as a sign of differentiation towards astrocytes; the latter adopts a more aggressive mesenchymal profile, known to be associated with shorter survival [43,44]. These differences may underlie the varying clinical outcomes seen in patients [43].

Further complexity arises from co-culture models. Advanced 3D bioprinting studies show that mesenchymal stromal cells, when co-cultured with glioma cells under both normoxic and hypoxic conditions, significantly boost the secretion and diversity of chemokines. These signals likely contribute to the spatial organization and immunological character of the GBM microenvironment [45].

Hypoxia also coexists with acidic stress in the tumour microenvironment, particularly in necrotic zones. Both hypoxia and low pH can independently influence gene expression and cellular behaviour in ways that promote GSC maintenance and tumour aggression [46].

Acidic environment is often caused by anaerobic glycolysis, a process in which tumour cells consume glucose and produce lactic acid. As a result, extracellular pH drops while oxygen tension plummets—conditions that do not occur in healthy tissues and which promote further tumour heterogeneity [46].

GSCs are typically identified using markers such as CD133, octamer-binding transcription factor 4 (OCT4), sex-determining region Y-box transcription factor 2 (SOX2) and B-cell-specific Moloney murine leukemia virus integration region 1 protein (BMI-1) [46,47]. CD133 is a cell surface protein that is involved in signal transduction via such classic molecular pathways as phosphatidylinositol 3-kinase (PI3K)/protein kinase B (Akt) and the wingless-related integration site (Wnt)/β-catenin cascade, finally correlating with stemness and self-renewal, as well as migration and invasion of the neoplastic cells [48]. SOX2 and OCT4 are transcription factors associated with proliferation, differentiation and self-renewal of cells. Both factors mutually interact to regulate gene expression via binding to DNA [49]. In malignant tumours, co-expression of SOX2 and OCT4 is a part of stem cell phenotype, associated with pluripotency, adverse prognosis and aggressive behaviour, characterized by high capacity to proliferate, migrate, and invade tissues [50]. BMI-1 is a member of polycomb group proteins, representing transcriptional inhibitors. BMI-1 participates in the regulation of cell proliferation, differentiation and senescence, as well as maintenance of the stem cell pool [47].

Exposure to hypoxia significantly increases the expression of these markers, supporting the theory that low oxygen enhances the stem-like phenotype of GBM cells. Interestingly, hypoxia also alters the glycosylation of CD133, which may affect its function and recognition in immunoassays. Other stemness markers, e.g., BMI-1, podoplanin, and nestin, are also upregulated in response to low oxygen. However, SOX2 expression appears to be more context-dependent, increasing only under specific 3D conditions that mimic in vivo environments [46].

In conclusion, a wide range of molecular responses to hypoxia—including HIF-driven transcriptional programmes, altered metabolism through enzymes like HK2, lncRNA-mediated feedback loops, and the activation of proteins such as galectin-8 and transgelin—demonstrates the remarkable plasticity of GSCs. These adaptations do not simply maintain GSC viability; they actively enhance their tumorigenic potential, allowing invasion, angiogenesis, and evasion of apoptosis. Notably, the interplay between hypoxia and inflammation, illustrated by RAGE signalling and necrosis-induced migration, highlights how environmental stressors converge to reinforce malignant progression.

Hypoxia also drives phenotypic diversity within gliomas, with IDH-mutant and IDH-wildtype tumours adopting distinct transcriptional profiles under low oxygen [51]. This observation reinforces the idea that hypoxia is a central determinant of glioblastoma heterogeneity, contributing to clinical variability and complicating treatment strategies.

Importantly, while standard therapies remain poorly effective against hypoxic tumour cores, the molecular pathways uncovered in recent years point toward promising therapeutic opportunities. Targeting HIFs, hypoxia-regulated lncRNAs and downstream effectors could disrupt the supportive hypoxic niche and sensitize GSCs to existing therapies. Moreover, the recognition that hypoxia coexists with acidic stress and immune modulation suggests that a combination of strategies aimed at dismantling these overlapping protective environments may be necessary to achieve durable responses.

Taken together, hypoxia is both the origin and the safeguard of GSC persistence. By positioning hypoxia as a central driver of glioblastoma’s resilience, future research can prioritize therapeutic approaches that specifically dismantle the protective adaptations fostered in these low-oxygen niches. Such strategies hold the potential to weaken the roots of glioblastoma recurrence and shift the treatment landscape for this devastating disease.

## 3. The Perivascular Niche—A Complex Arena of GSC-Endothelial Interactions

While hypoxia supports the survival of GSCs through metabolic and genetic adaptations, another critical component of the microenvironment in glioblastoma is the perivascular niche (PVN). This region, found adjacent to the tumour vasculature, is home to a dense network of cellular interactions between endothelial cells, mesenchymal cells, pericytes, and GSCs. The PVN not only supports GSC maintenance through direct signalling but also participates in the development of treatment resistance and promotes tumour growth. In this section, we explore the cellular complexity of the perivascular regions, the molecular mechanisms taking place within them, and the implications for potential therapeutic interventions.

Microvascular proliferation (MVP), a defining histological hallmark of GBM, has traditionally been associated with the activity of endothelial cells (ECs). However, transcriptomic analyses have expanded our understanding by revealing that mesenchymal-like perivascular stromal cells (PVSCs)—such as pericytes, connective tissue cells and mesenchymal stem cells—are also central players in MVP. These cells engage in intricate communication with immunosuppressive myeloid cells, indicating a multilayered complexity within the vascular niche and highlighting receptor-ligand interactions that may serve as novel therapeutic targets [52].

GSCs often cluster near blood vessels within the perivascular niche, where they receive instructive signals that preserve their stem-like state. However, endothelial cells may exert not only supportive but also inhibitory effects. For example, SEMA3G—a semaphorin secreted by endothelial cells—has been shown to suppress GSC characteristics by triggering *c-Myc* degradation through the neuropilin 2 (NRP2)/plexin-A1 (PLXNA1)—cell division control protein 42 homologue (Cdc42)—WW domain containing E3 ubiquitin protein ligase 2 (WWP2) signalling axis. SEMA3G binds to its receptor NRP2/PLXNA1, leading to activation of intracellular GAP domain of PLXNA1. The next molecular event is inactivation of Rho-GTPase Cdc42. Activated Cdc42 would bind E3 ubiquitin ligase WWP2, preventing ubiquitination of *c-Myc* by this ligase. In contrast, inactive CdC42 does not bind WWP2 and *c-Myc* undergoes ubiquitination and is destroyed. As *c-Myc* is necessary to maintain stemness via PI3K/AKT molecular pathway, loss of this protein helps to decrease stem cell differentiation in glioblastoma. This identifies SEMA3G as a potential anti-stemness signal that could be leveraged therapeutically [53].

Adding to the heterogeneity of endothelial populations, recent findings reveal the presence of lymphatic endothelial-like cells (LECs) within GBM—previously unrecognized in normal brain tissue. These LECs promote the proliferation of C-C chemokine receptor type 7 (CCR7)-positive GSCs through the secretion of the chemokine (C-C motif) ligand 21 (CCL21). Interruption of the CCL21–CCR7 axis markedly impairs GSC growth. Mechanistically, CCL21 stabilizes 3-hydroxy-3-methylglutaryl-CoA synthase 1 (HMGCS1), a key enzyme in cholesterol synthesis, via lysine acetyltransferase 5 (KAT5)-mediated acetylation. Acetylation increases the stability of HMGCS1 protein, and stabilized HMGCS1 promotes cholesterol synthesis in GSCs, which is favourable for tumour progression [54]. Furthermore, we explore the newest research models for studying the perivascular niche of glioblastoma.

Integrative approaches using RNA sequencing and spatial transcriptomics have revealed niche-specific gene expression patterns in GBM. For instance, regions around necrosis (pseudopalisading areas) and microvascular zones show differential expression of N-myc-downstream regulated gene-1 (*NDRG1*) and endothelial PAS domain protein 1 (EPAS1) known also as HIF-2alpha. *NDRG1* is associated with cell migration, angiogenesis and drug resistance in glioblastoma, while EPAS1/HIF-2alpha mediates response to hypoxia. Moreover, endothelial cells in these zones demonstrate genomic alterations that mirror those of glioblastoma cells, suggesting differentiation of GSCs into endothelial-like phenotypes [55].

Within the hypoxic perivascular niche, specialized cells expressing both nestin and CD31 dominate in microvascular structures and correlate with poor prognosis in GBM patients. These cells, arising from the differentiation of glioblastoma stem-like cells, can promote drug resistance by increasing the production of Jagged1 (JAG1) and delta-like canonical Notch ligand 1 (DLL4)—ligands that bind to their receptors Notch-1 and Notch-2, activating the Notch molecular cascade, which in turn leads to activation of the downstream target genes *Hes1* and *Hey1* [56]. The summary effect of the activation of Notch molecular pathway is promotion of glioblastoma stem cells. In contrast, blockade of the Notch pathway results in reduced expression of stemness markers as well as inhibition of neurosphere formation in vitro [56]. Increased expression of the transcription factor Hes1 serves as evidence of the activation of Notch cascade; thus, an association with adverse prognosis via increased stemness can be expected. Notably, it is the Hes1 expression in tumour cells within the hypoxic perivascular niche, not the total tumour tissue, that predicts poor clinical outcomes [57,58].

One more innovative 3D in vitro model of the perivascular niche, developed by R. Hatlen et al., integrates human umbilical vein endothelial cells (HUVECs) and GBM cells (LN229) within a biomaterial environment. Over time, this model demonstrated significant matrix remodelling, upregulated signalling via chemokine pathways (characterized by, e.g., elevated levels of C-X-C motif chemokine ligand CXCL12 and transforming growth factor-beta TGF-β), and a dramatic increase in cellular proliferation. In addition, a significant fraction (at least 15%) of glioblastoma cells started to co-express endothelial immunohistochemical markers, e.g., von Willebrand factor, showing transdifferentiation to endothelium [59]. In a xenograft mouse model, injected with human glioblastoma stem cells, most of the central blood vessels in the tumour are of human origin, further proving the transdifferentiation. Importantly, this process depends on the presence of stem cells in glioblastoma, not bulk of tumour [60]. Besides transdifferentiation to endothelium, a fraction of glioblastoma stem cells undergo transdifferentiation into pericytes [61]. Consequently, the blood vessels in glioblastoma are of mixed reactive and neoplastic composition, limiting the possibilities of anti-angiogenetic therapy. These findings underscore how tumour–vascular interactions actively reshape the physical architecture, reflected both by cellular transdifferentiation in blood vessels and hydrogel contraction in the experimental conditions, and signalling within the tumour microenvironment [59].

Another advanced platform—a perivascular niche-on-a-chip developed by Gerigk et al.—replicates in vivo conditions using a 3D extracellular matrix and gravity-driven flow to support co-culture of endothelial cells from multiple tissue origins with patient-derived GSCs. The model preserved tight junction integrity and vascular identity in serum-free conditions and revealed upregulation of angiogenesis-related genes when GSCs were present, compared to neural stem cells. This system provides a powerful tool for dissecting tumour–vessel dynamics in real-time [62].

The perivascular niche represents a dynamic and multifaceted domain in which glioblastoma stem cells receive critical survival cues, adapt to environmental pressures, and even influence endothelial behaviour. With the discovery of niche-specific molecular interactions—ranging from SEMA3G-mediated suppression to CCL21-driven metabolic support—the PVN emerges as both a sanctuary and a battlefield for GSC regulation. Targeting these interactions offers a promising frontier for GBM therapy, especially in combination with strategies that simultaneously address hypoxia and immune modulation.

## 4. Immune Modulation and Inflammatory Interplay

Beyond the physical scaffolds of hypoxia and vasculature, the glioblastoma tumour microenvironment (TME) is deeply shaped by immune interactions. Glioblastoma stem cells (GSCs) not only survive in this hostile immunological landscape but also manipulate it to suppress anti-tumour immunity and enhance tumour-promoting inflammation.

Central to this manipulation are tumour-associated macrophages (TAMs) [63], regulatory T cells (Tregs) [64], and other immune players that are reprogrammed or disabled in the presence of GSC-secreted factors. In this section, we examine how GSCs reshape immune activity to sustain growth, and we highlight new findings in immune regulation, resistance to checkpoint blockade, and the emerging promise of targeted immunotherapy.

**Figure 2:**
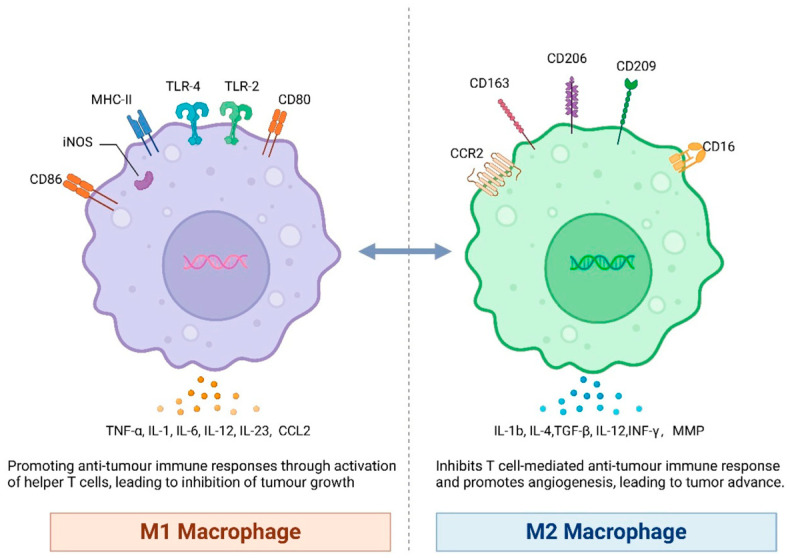
Two types of macrophages: M1 promoting anti-tumour response and M2 promoting angiogenesis and, respectively, tumorigenesis. Copyright © 2024 by the authors Li, Ye and Luo. Licensee MDPI, Basel, Switzerland. Figure replicated from M.-Y. Li et al. [66] under the terms and conditions of the Creative Commons Attribution License (CC BY), provided at https://creativecommons.org/licenses/by/4.0/ (accessed on 1 February 2026). Changes made: Figure legend.

Glioblastoma stem cells actively alter the immune microenvironment by attracting and reprogramming tumour-associated macrophages (TAMs) into an immunosuppressive M2-like state. This polarization is driven by cytokines and chemokines such as macrophage colony stimulating factor (M-CSF),transforming growth factor beta 1 (TGF-β1), and interleukin 10 (IL-10), along with immune checkpoint molecules including cluster of differentiation 47(CD47) and B7 homolog 4 (B7H4). Collectively, these signals impair macrophage phagocytosis and promote immune evasion, enabling tumour progression with reduced immune interference [65], Figure 2.

In a study by Zhang et al., researchers used CRISPR-Cas9 and gene overexpression techniques to explore the role of the transcription factor nuclear factor erythroid-2-like 1 (NFE2L1). NFE2L1 participates in the maintenance of mitochondrial homeostasis, including the mitochondria of macrophages. In the M1 versus M2 polarization of macrophages, NFE2L1 suppresses the formation of M1 macrophages. Consequently, in gliomas, NFE2L1 is a key player, promoting the pro-tumourigenic M2 phenotype of tumour-associated macrophages. Importantly, inhibition of NFE2L1 was shown to reverse immune suppression and could enhance the efficacy of immunotherapy. This suggests that targeting NFE2L1 may be a valuable strategy in future combination treatments [67].

GSCs also secrete a spectrum of chemokines—such as CCL2, CCL5, CCL7, CX3CL1, and vascular endothelial growth factor A (VEGF-A)—that enhance TAM recruitment and M2 polarization. This chemokine-driven inflammation promotes a tumour-supportive microenvironment and represents a critical axis for therapeutic disruption [65]. 

Beyond macrophages, the GBM microenvironment includes a diverse array of immune cells.

Regulatory T cells (Tregs): These cells suppress immune responses by releasing IL-10 and TGF-β and are recruited through chemokines like CXCR3 and CCR5 [68]. Attempt to activate anti-cancer qualities of TME in glioblastoma was made by Johnson et al.

Generally, glioblastoma stem cells utilize mechanisms of immunosuppression and tend to inhibit cytotoxicity towards tumour cells [69]. Although a multifaceted study showed immunosuppressive action of Treg-like immunosuppressive cells towards the mesenchymal subset of GSCs [69].

Natural killer (NK) cells: Normally potent tumour killers, NK cells are inhibited in GBM through TGF-β signalling and molecular mimicry of major histocompatibility complex class I (MHC-I), which blunts their cytotoxicity [68]. Nevertheless, there are studies which have shown the possibility of NK cells to attack cancer-propagating cells [70]. Image-based assay, made by Du et al., examined NK cell migration and cytotoxicity towards GSCs. This study represents a useful methodology for future NK cell-derived anti-cancer strategies [71].

Dendritic cells (DCs): While capable of initiating anti-tumour immunity, DCs in GBM are often poorly recruited and functionally impaired, though clinical trials using DC vaccines show promise [68]. Several studies made in the past showed promising results and safety of autologous tumour lysate-pulsed therapy [72,73].

In the present-day, studies show significant clinical activity of autologous dendritic cell vaccine against a variety of solid tumour including glioblastoma [74].

Neutrophils: Attracted by IL-8, these cells paradoxically enhance tumour invasion and blood vessel formation via NF-κB signalling. Elevated neutrophil-to-lymphocyte ratios (NLRs) are associated with worse outcomes and may outperform traditional markers like IDH1 mutation in prognosis [68]. Neutrophil recruitment towards tumour niche amplifies necrosis and leads to progression of tumour [75]. Targeting of this immune cell population is one more promising objective for glioblastoma immune therapy. Examples of anti-neutrophil action mechanisms include neutrophil mediated macrophage induced tumour cell phagocytosis [76], TGF-β blockage which results in anti-tumorigenetic properties of neutrophils [77], TRAIL-mediated neutrophil apoptosis [78], and many others.

To further understand immune dynamics in glioblastoma, Mongeon et al. constructed a mathematical model incorporating CD8+ T cells, pro- and anti-tumoral TAMs, and clinical therapies such as standard of care (SOC) and the programmed cell death protein 1 (PD-1) immune checkpoint inhibitor nivolumab. The model revealed that PD-1 blockade is often ineffective due to poor recruitment of CD8+ T cells, largely as a result of TAM-driven immunosuppression. However, modifying TAM function—particularly by restoring their phagocytic ability through anti-CD47 therapy—led to near-eradication of tumours in silico. This underscores that rather than simply depleting TAMs, reprogramming their function may yield significant therapeutic benefit [79].

In addition, Adjei-Sowah et al. developed a microfluidic triculture model mimicking the perivascular niche to investigate GSC behaviour in the presence of astrocytes and endothelial cells. Both supporting cell types were found to increase GSC invasiveness. Single-cell RNA sequencing identified 15 ligand–receptor pairs that may mediate chemotactic migration of GSCs. Among them, the ligand–receptor pair of serum amyloid A 1 (SAA1) and its receptor, G protein-coupled formyl peptide receptor 1 (FPR1) stood out for its role in directing GSCs toward blood vessel-rich regions. This model offers a sophisticated platform for drug testing and mechanistic studies [80].

Further exploration of perivascular interactions revealed that glioma vascular cells (GVCs) and tumour cells (GTCs) communicate through secreted factors. Using RNA sequencing and functional assays, researchers identified integrin-binding sialoprotein (IBSP) as a key molecule promoting tumour growth and migration along blood vessels. The resulting interactome map could serve as a foundational resource for targeting GSC–vascular communication in GBM [81].

To summarize, the immunological landscape of glioblastoma is carefully shaped by glioblastoma stem cells, which use cytokines, checkpoints, and chemokines to reprogram immune responses in their favour. Rather than being passive targets, immune cells—especially TAMs—are actively co-opted into supporting the tumour. As immunotherapy continues to evolve, strategies that reshape these interactions—such as targeting CD47, NFE2L1, or the chemokine axes—hold enormous potential. By converting the immune microenvironment from a sanctuary into a battlefield, future treatments may finally breach glioblastoma’s deepest lines of defence.

## 5. Summarizing Interactions Between Hypoxic, Vascular and Immune Niches Driving Glioblastoma Stem Cells and Tumorigenesis

In this section we want to summarize and highlight interactions in which hypoxia, vasculature and immune modulation are working together to upregulate glioma stem cells and promote tumorigenesis.

We mentioned how Gassmann et al. in the 90s highlighted upregulation of expression of endothelial growth factor (VEGF) under hypoxic conditions in embryonic stem cells [13]. VEGF, presumably, is promoting glioblastoma by stimulating GSC proliferation via its respective receptor and subsequent vascular proliferation in the tumour [82]. Hypoxia-inducible factors (HIFs) also regulate VEGF expression and stemness of glioblastoma, as was stated by Schito and Semenza [22]. Hypoxia also showed itself as an angiogenesis promoting factor in 3D cultures from human tumours [43].

Chen et al. highlighted how hypoxic conditions affect immune cells in glioblastoma microenvironment. Respectively, tumour-associated macrophages TAMs secreting TGFBI under hypoxic conditions, preventing EphA2 oncogene-coded protein degradation [38]. 3D bioprinting also showed multiple chemokine expression in glioblastoma perivascular niche, connecting immune and vascular components of the tumour microenvironment [45]. TAMs also, supposedly, upregulate tumour angiogenesis [83]. Vascular proliferation is a substantial hallmark of glioblastoma [52] and driver of its aggressivity and stemness. Therefore, other tumour microenvironment components and interactions commonly lead towards perivascular niche.

Lymphatic endothelial-like cells are one more interesting entity connecting glioblastoma perivascular niche and immune system. These cells upregulate proliferation of CCR7-positive GSCs through the secretion of the chemokine CCL21, leading to more effective tumorigenesis. Disruption of this process represents one more promising therapeutic target [54].

It also important to highlight once more GSC interactions with immunologically active astrocytes and endothelial cells, leading to chemotactic migration of GSCs near perivascular niche [80]. As well as vascular cell and glioblastoma tumour cell communication via secreted molecules [81].

Together, these findings demonstrate that hypoxia, vascular structures, and immune cells act cooperatively to create a supportive microenvironment that sustains GSCs and drives glioblastoma progression. Targeting these interconnected pathways may provide promising therapeutic strategies to disrupt the perivascular niche and limit tumour growth and recurrence.

## 6. Conclusions

Glioblastoma remains one of the biggest challenges in oncology, owing to its remarkable ability to adapt, invade, and resist conventional therapies. At the core of this resilience lies the glioblastoma stem cell (GSC) population, which thrives in specialized microenvironments that both shelter and empower it. Across hypoxic regions, perivascular zones, and immune-modulated compartments, GSCs engage in dynamic interactions that collectively reinforce tumour heterogeneity, aggressiveness, and therapeutic resistance.

Hypoxia emerges as a defining hallmark of glioblastoma biology, where oxygen deprivation fuels metabolic reprogramming, activates hypoxia-inducible factors, and drives a suite of molecular adaptations that strengthen stemness and survival. The perivascular niche represents a second critical axis of support, where endothelial and stromal cells provide instructive cues that can either sustain or, in rare cases, suppress GSC identity. Finally, the immune landscape—dominated by reprogrammed macrophages, impaired T and NK cells, and tumour-promoting inflammation—illustrates how GSCs do not merely withstand immune pressure but actively reshape it to their advantage.

Taken together, these findings underscore glioblastoma not as a homogeneous mass of rapidly dividing cells, but as a structured ecosystem where GSCs act as architects of persistence. Importantly, no single niche acts in isolation. Hypoxia intersects with vascular remodelling; vascular cues converge with immune suppression; and immune modulation feeds back into hypoxia and angiogenesis. This network of reinforcing pathways explains why glioblastoma is so resistant to therapies and highlights the urgent need for integrated strategies.

The therapeutic implications are profound. Future interventions must move beyond targeting proliferating tumour cells and instead disrupt the supportive niches that enable GSC survival. HIF inhibitors, modulators of endothelial–GSC signalling, and agents that reprogram tumour-associated macrophages look promising. Particularly when combined with established modalities such as chemotherapy, radiotherapy, or immune checkpoint blockade. The development of advanced 3D culture systems, organoid models, and niche-on-a-chip technologies provides unprecedented opportunities to test such approaches in physiologically relevant contexts.

In conclusion, understanding glioblastoma through the lens of its stem cell populations and their microenvironments reframes the disease not simply as uncontrolled proliferation but as a highly adaptive ecosystem. By dismantling the reciprocal support systems across hypoxia, vasculature, and immune modulation, future therapies may finally erode the foundations of GSC-driven resilience. Such a paradigm shift holds the greatest promise for extending survival and, ultimately, altering the trajectory of this devastating disease.

## Data Availability

No new data were created or analyzed in this study. Data sharing is not applicable to this article.
